# National screening for delirium in paediatric intensive care units: A quality improvement initiative

**DOI:** 10.1111/nicc.13303

**Published:** 2025-02-24

**Authors:** Bronagh Blackwood, Leanne M. Aitken, Jennie Craske, Sandra Gala‐Peralta, Ashley Liew, Maeve Murray, Lisa McIlmurray, Lyvonne N. Tume

**Affiliations:** ^1^ Wellcome‐Wolfson Institute for Experimental Medicine Queen's University Belfast Belfast UK; ^2^ School of Health & Psychological Sciences, City University of London London UK; ^3^ Alder Hey Children's Hospital NHS Foundation Trust Liverpool UK; ^4^ Royal Brompton Hospital Guy's and St Thomas Hospital NHS Foundation Trust London UK; ^5^ Department of Children's Neurosciences, Evelina London Children's Hospital Guys and St Thomas' NHS Foundation Trust London UK; ^6^ National & Specialist CAMHS South London and Maudsley NHS Foundation Trust London UK; ^7^ Centre for Educational Development Appraisal and Research (CEDAR) University of Warwick Coventry UK; ^8^ Institute for Mental Health University of Birmingham Birmingham UK; ^9^ Women Children and Families Division Antrim Area Hospital, Northern Health & Social Care Trust Antrim UK; ^10^ Paediatric Intensive Care Unit Children's Health Ireland at Temple Street Dublin Ireland; ^11^ Faculty of Health, Social Care & Medicine Edge Hill University Ormskirk UK

**Keywords:** child, critical care, delirium, quality improvement

## Abstract

**Background:**

Internationally, one in three children develop delirium during their intensive care stay. International guidelines strongly recommend twice‐daily screening for paediatric delirium using validated instruments. In the United Kingdom and Ireland, delirium was assessed only when suspected and few intensive care units (ICUs) used validated instruments.

**Aim:**

This initiative aimed to implement a national screening strategy in 28 paediatric intensive care units (PICUs) across the United Kingdom and Ireland.

**Study Design:**

The strategy involved: (a) rapidly reviewing, evaluating and ranking paediatric screening instruments for sensitivity, specificity, appropriateness and acceptability for national implementation; (b) achieving national agreement to implement a common tool; (c) creating and disseminating training materials while supporting training personnel in implementation; and (d) integrating delirium monitoring within the Paediatric Intensive Care Audit Network national database.

**Results:**

Among seven validated instruments, the top ranked options (from 1, most applicable to 7, least applicable) were the Cornell Assessment of Pediatric Delirium (average rank 1.25) and the Sophia Observation withdrawal Symptoms‐Paediatric Delirium scale (1.5). Twenty‐three units voted for their preferred choice of instrument: fifteen preferred the Cornell instrument, eight favoured the Sophia instrument and five did not respond. Training and implementation began in November 2021 and by March 2023 18 of the 28 units (64%) had successfully implemented screening. The national database began actively collecting delirium data from units in January 2024.

**Conclusions:**

This initiative outlined critical steps for implementing and maintaining practice of delirium screening in PICUs. We provided clinicians with validated screening tools for detecting paediatric delirium and the necessary support and infrastructure to maintain screening. Embedding and sustaining screening is an ongoing challenge.

**Relevance to Clinical Practice:**

Undertaking routine screening for all intensive care patients from admission to discharge using validated instruments will provide earlier detection and treatment for critically ill children. This strategy offers a model for standardized and effective implementation in clinical practice in ICUs.

**What is known about the topic**

- Delirium is an acute dysfunction of the brain in the setting of critical illness. It is highly distressing, characterized by attention and cognitive deficits, hallucinations and fluctuating consciousness. In children, delirium is under‐recognized and misunderstood, largely due to lack of screening. One in three children develop delirium during their intensive care stay. International guidelines strongly recommend twice‐daily screening for paediatric delirium using validated instruments. In the United Kingdom and Ireland, delirium was assessed only when suspected and few intensive care units used validated instruments.

**What this paper adds**

- This paper reports a comprehensive national plan for implementing screening using validated instruments for detecting paediatric delirium. The national screening strategy will provide many benefits. It will enable delirium incidence to be monitored effectively over time, facilitate comparison of outcomes in future interventions designed to mitigate delirium, and more importantly, will allow earlier detection and treatment for critically ill children.

## 1 INTRODUCTION AND BACKGROUND

Delirium is defined as a noticeable change in a person's neurocognitive baseline with an acute disturbance in their attention, awareness and cognition.^1^ The change may be classified into hyperactive (agitated) and hypoactive (lethargic) subtypes or a mixed form of both presentations. In the paediatric intensive care unit (PICU), delirium may result from metabolic and clinical factors that include infections, derangement in electrolytes, anaemia, sedative and opioid withdrawal, sleep disturbance and sensory overload. Delirium is a severe complication of PICU admission that can result in increased mortality, morbidity and hospital costs because of increased length of stay.^2^, ^3^

Delirium is purported to be relatively common in children admitted to the PICU, and yet under‐reported in comparison to adult literature.^2^, ^3^, ^4^ An international prevalence study reported 34%,^5^ but due to inconsistent screening for paediatric delirium worldwide, the prevalence is likely to be under‐estimated.^5^, ^6^, ^7^ Prevalence rates of paediatric delirium have been retrospective and from single sites; thus, generalization is difficult.^5^

### 1.1 Rationale for the quality initiative

Assessing delirium using validated instruments can significantly increase and improve detection in the intensive care unit (ICU).^8^ Indeed, European position papers and American guidelines strongly recommend daily screening for paediatric delirium using validated instruments.^9^, ^10^ Within UK and Ireland PICUs, regular screening for delirium was not conducted, resulting in a lack of data of prevalence rates, evident from Semple et al.^5^ systematic review of studies reporting prevalence rates. Despite this, there is substantial interest in, and momentum around, paediatric delirium fuelled by prioritization of delirium in research as a key concern by the UK Paediatric Critical Care Society (PCCS) and parents.^11^ In June 2020, the PCCS research group met to discuss the direction of research in delirium. The lack of screening allowed many PICUs to adopt a common delirium screening instrument. This would ensure consistent delirium measurement in research, development of shared training instruments and detection for timely treatment and prevention. In July 2020, a paediatric delirium group for the United Kingdom and Ireland (PDGUKI) was established and a multidisciplinary core team developed an implementation strategy for delirium screening. The main aim of this national initiative was to implement a national screening strategy in 28 PICU across the United Kingdom and Ireland.

**FIGURE 1:**
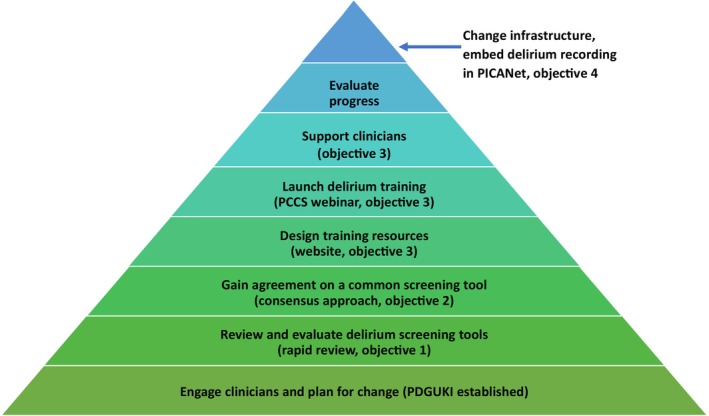
Implementation strategy model based on the Expert Recommendations for Implementing Change.^12^

Figure 1 offers a comprehensive visual representation of the initiative described in this paper. The implementation strategy was based on the Expert Recommendations for Implementing Change [ERIC].^12^ At the bottom of the pyramid the model shows the initial step of engagement (establishing the PDGUKI). The next steps reflect the initiative's objectives that include, selecting and reaching consensus on a common screening tool, developing the training resources, launch of the initiative and support process. At the top of the pyramid the model indicates how a change in infrastructure (initiating daily data collection via the national database) can facilitate sustained change.

## 2 AIM AND OBJECTIVES

The aim of this national initiative was to implement a national screening strategy in 28 PICU across the United Kingdom and Ireland. The objectives were to:

1. Rapidly review, evaluating and rank paediatric screening instruments for sensitivity, specificity, appropriateness and acceptability for national implementation
2. Achieve national agreement to implement a common tool
3. Creating and disseminate training materials and support training personnel in implementation
4. Integrating delirium monitoring within the Paediatric Intensive Care Audit Network (PICANet) national database.

## 3 DESIGN AND METHODS

This was a quality improvement initiative conducted in the PICUs in the United Kingdom and Ireland, and it is reported using the SQUIRE reporting guidelines.^13^ The methods employed to achieve each objective are outlined below.

### 3.1 Rapid review of paediatric delirium screening instruments

This rapid review used modified systematic review methods to accelerate the review process while maintaining systematic and transparent methods.^14^

#### 3.1.1 Review question

Which screening instrument best predicts delirium in infants and children and is feasible to administer by clinical practitioners?

#### 3.1.2 Inclusion criteria

Included papers:

- Focused on infants and children from birth to 16 years in the PICU
- Included instruments or questionnaires used for screening paediatric delirium
- Reported on a single instrument screening instrument
- Reported on instrument development and reliability and validity testing
- Published in English from the year 2000 as there were limited publications in paediatric delirium prior to this

Excluded papers:

- Were not single instruments (i.e., they included a battery of neuropsychological tests)
- Could not be administered by clinical staff (i.e., required specialized psychiatric training).

#### 3.1.3 Search strategy and data extraction

OVID Medline, Cumulative Index to Nursing and Allied Health Professionals and PubMed were searched from 1 January 2000 to 31 August 2020 prior to planning the initiative. Search terms included critical or intensive care, paediatric, delirium, screening tools or instruments and psychometrics. (Supplement, Search strategy) Reference lists of included studies were also searched. One reviewer (BB) screened titles, abstracts and full texts for study inclusion. A second reviewer (LT) checked study inclusion.

Data were extracted from the original instrument papers and, if relevant, papers reporting subsequent versions. Data were independently extracted by reviewers (AL, BB, LA, LT) concerning the instrument's predictive power (sensitivity, specificity and area under the curve [AUC]); ability to cover all delirium subtypes [hyper/hypoactive and mixed]; age range suitability; and implementation feasibility (items, time to complete and other). Data were not extracted from papers reporting subsequent translation and validation or reliability testing in various countries.

#### 3.1.4 Data analysis

Each reviewer (AL, BB, LA, LT) independently ranked the screening instrument's overall suitability for use in PICUs based on the instrument's predictive power, ability to cover all delirium subtypes, age range suitability and implementation feasibility. Instruments were ranked from 1 (most applicable) to 7 (least applicable) and reasons were recorded to justify the ranking. The rank scores for each instrument were summed, averaged and tabulated by two reviewers (BB, LA).

### 3.2 Consensus method to achieve national agreement to implement a common tool

The consensus methods included two virtual meetings of the PDGUKI members. Meetings were conducted on Zoom.

At the first virtual meeting a presentation of the rapid review results was provided by the review team. The presentation was followed by an open discussion to clarify the finding and address questions. Members were tasked to return to their units and discuss the review findings, to obtain their unit's preferred choice of screening instrument, and to propose a representative to lead communication, training and implementation of delirium screening in their unit.

At the second virtual meeting the training representative (or nominated person) was asked to provide their unit's vote for their preferred choice of a common instrument. Voting was conducted using a poll on Zoom and only one vote per unit was accepted. Those unable to attend the meeting were requested to send their votes to the PDGUKI chair by email.

Data were analysed by counting the agreed vote delivered by the unit's nominated person.

### 3.3 Creating and disseminate training materials and supporting training personnel

Given the challenges of implementing delirium screening across five countries, appropriate implementation methods were used to guide the design, delivery and embedding of screening. A core team (the authors) was established to design training materials for screening implementation using a multifaceted strategy combining passive and active interventions.^15^, ^16^ Passive strategies included developing educational materials, posters, toolkits and visual aids, while active strategies focused on engagement through workshops, feedback audits and reminders.

In planning delivery of the training materials and support, the team was guided by the nine themes identified in the ERIC study.^12^ The ERIC study addressed the inconsistency in guidance from the myriad of implementation strategies for developing and planning implementation initiatives. Essentially the study mapped all the available strategies into themes that provided a core set of concepts in implementation science. The ERIC themes addressed engaging consumers, using evaluative and iterative strategies, changing infrastructure, adapting and tailoring the context, developing stakeholder interrelationships, supporting clinicians, providing interactive assistance, training and educating stakeholders and utilizing financial resources.

To disseminate training resources to support training personnel, the team collaborated with the Department of Computer Science to plan a dedicated PDGUKI website. The website would include a number of web pages that would hold training resources, audit and feedback charts, pre‐ and post‐training questionnaires and certificates, dates of monthly PDGUKI meetings and relevant news. Furthermore, plans were made to hold a PCCS Webinar to raise the impact of paediatric delirium and launch the website and training materials.

### 3.4 Integrating delirium monitoring within PICANet

Planning to achieve integration included holding a series of discussions with the PICANet Clinical Advisory Group (CAG) to discuss embedding mandatory daily delirium data collection in their database for all PICU admissions. PICANet incorporates 28 PICUs in the United Kingdom and Ireland.^17^ Each PICU contributes demographic and clinical data on all admissions to the PICANet database. These data are used to monitor supply and demand, outcomes, planning and resource requirements and to study epidemiology of critical illness. Once agreed by the PICANet CAG, further discussions were held with the technical staff to provide the dataset definition and outline data requirements prior to publishing the information in the PICANet Dataset Manual.

## 4 ETHICS: SERVICE USER INVOLVEMENT AND APPROVALS

PICU staff and parents were instrumental in prioritizing delirium through the prioritization process.^11^ Staff were engaged in all steps of the process. Ethical approval was not required for this quality improvement initiative and the national PCCS and PICANet approved this initiative.

## 5 RESULTS

### 5.1 Rapid review results

#### 5.1.1 Search

**FIGURE 2:**
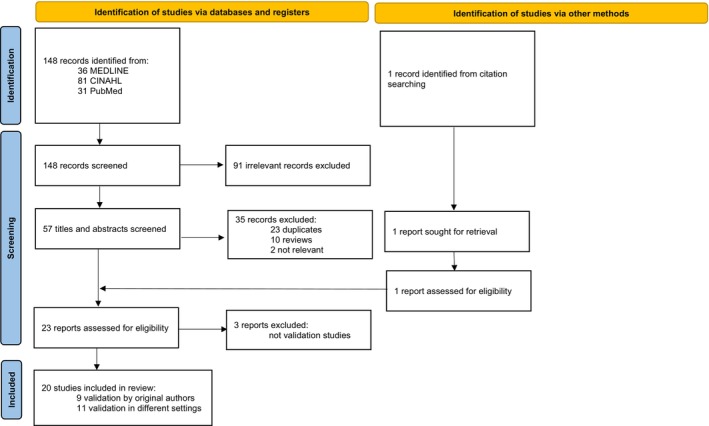
Flowchart of Studies included in the Review.

From 148 citations, 22 papers and 1 citation from references were identified. On full‐text review, three papers that were not instrument validation studies were excluded. Twenty papers were included in the review (Flowchart, Figure 2).

#### 5.1.2 Included studies

Of 20 papers, nine reported instrument development, original validation and/or adaptations for seven delirium screening instruments.^18^, ^19^, ^20^, ^21^, ^22^, ^23^, ^24^, ^25^, ^26^ Eleven papers reported further validation of the instruments in international settings.^27^, ^28^, ^29^, ^30^, ^31^, ^32^, ^33^, ^34^, ^35^, ^36^ The seven instruments and their characteristics, and the associated validation studies are shown in Table 1.

**TABLE 1:** Characteristics of paediatric delirium screening instrument studies in chronological order of development.

Tool	Original development versions and adaptations	Validations	Original criterion	Sensitivity	Specificity	AUC	Delirium Subtypes	Age range	Items/time	Item domains
Delirium Rating Scale (DRS‐88)	2003 Turkel	2011 Janssen	Psychiatrist DSM‐III‐R criteria	91.7%	100%	Item range 0.76–0.9. Cognition item range 0.45–52	NR	Mean 7‐years (SD 5.2)	Ten items Time NR	Cognition Mood Sleep–wake Behavioural
Pediatric Assessment Emergence Delirium (PAED)	2004 Sikich	2009 Bong, Asia; 2011 Janssen	DSM‐III‐R criteria	ROC True positive rate 0.64	ROC False positive rate, 1 minus 0.14	ROC AUC 76.6%	Emergence, hyperactive	Designed for 18‐months to 6‐years	Five items Time NR	Behavioural
Paediatric Confusion Assessment Scale for ICU (pCAM‐ICU)	2011 Smith	2020 de Castro, Brazilian Portuguese	Psychiatrist DSM‐IV criteria	83% (95% CI 66–93) IMV 75% (95% CI 66–100)	99% (95% CI 95–100) IMV 92% (95% CI 67–100)	NR	NR	Designed for 5‐years and older	Four steps Time 2‐min	Neurocognitive
Cornell Assessment Paediatric Delirium (CAPD)	2012 Silver 2015 Silver, added anchor points 2020 Kaur, added RASS	2014 Traube, USA; 2019 Simeone, Italy; 2020 Simonsen, Denmark; 2019 He, China; 2019 Valdivia, USA; 2020 Hoshino, Japan	Psychiatrist DSM‐IV criteria	92% (95% CI 85.7–98.3)^a^ 96.2% (95% CI 86.5–100)^b^	86.5% (95% CI, 75.4–97.6)^a^ 51.2% (95% CI, 24.7–77.8)^b^	0.96^a^ 0.86^b^	Hypoactive Hyperactive Mixed	Designed for birth to 13‐years	Eight items Time 2‐min	Behavioural Cognition
Pre‐school Confusion Assessment Scale for ICU psCAM‐ICU)	2016 Smith	2019 Matsuishi, Japan	Psychiatrist DSM‐IV criteria	75% (95% CI 72–78)^c^ 78% (95% CI 75–80)^d^	91% (95% CI 90–93)^c^ 86% (95% CI 84–88)^d^	NR	Hypoactive Hyperactive Mixed	Designed for 18‐months to 6‐years	Four steps Time 2‐min	Neurocognitive
Severity scale Paediatric Confusion Assessment Scale for ICU (sspCAM‐ICU)	2016 Luetz	None	Psychiatrist DSM‐IV criteria	71.8%	96.6%	NR	NR	Designed for 5‐years and older	Four steps Time 2‐min	Neurocognitive
Sophia Observation Scale and Paediatric Delirium (SOS‐PD)	2018 Ista	2018 Ista, multicentre	Psychiatrist if score greater than three on two occasions DSM‐IV criteria	96.8% (95% CI 80.4–99.5)	92.0% (95% CI 59.7–98.9)	NR	NR	Median 49‐months (IQR 13, 140)	Twenty two items Time NR	Behavioural Parental views

*Note*: *Results taken from its first test in paediatric ICU population by.^28^

Abbreviations: AUC, area under the curve; CI, confidence interval; DSM, Diagnostic Statistical Manual; IMV, invasive mechanical ventilated; NR, not reported; RASS, Richmond Agitation‐Sedation Scale; ROC, receiver operating characteristic; USA, United States America.

^a^ Normal development.

^b^ Developmental delay.

^c^ Long research version.

^d^ Short clinical version.

The reference standard for all instruments was the psychiatric review of delirium diagnosis, using the version of the Diagnostic and Statistical Manual^1^ applicable at the time of each study. Among the seven instruments, six were developed in North America,^20^, ^21^, ^23^, ^24^, ^25^, ^26^ while the Sophia Observation Scale and Paediatric Delirium scale (SOS‐PD) originated in the Netherlands.^18^

The earliest instrument, the Delirium Rating Scale (DRS‐88), was created in 1988 for adults.^37^ In 2003, the DRS‐88 was tested using retrospective chart review in a cohort of ‘seriously ill’ children, but not specifically for PICU.^26^ In 2011, the DRS was validated for use in the PICU.^28^ Consisting of 10 items, it assesses cognition, mood, sleep‐awake cycle and behaviour.

The Pediatric Assessment of Emergence Delirium (PAED) instrument was developed in 2004 to specifically detect mental disturbances during the recovery from general anaesthesia.^21^ Using five items, this instrument focuses on assessing behavioural manifestations of mental disturbance. Emergence delirium is typically short‐lived in the post‐operative paediatric population.

Three paediatric versions of the adult Confusion Assessment Scale for ICU (CAM‐ICU)^38^ were adapted to assess neurocognitive symptoms in age‐appropriate ways. They include the psCAM‐ICU for children aged 18 months to 6 years,^25^ pCAM‐ICU for children older than 5 years^24^ and sspCAM‐ICU for measuring severity on a scale from 0 to 19.^20^

The Cornell Assessment of Pediatric Delirium (CAPD) instrument^23^ built upon the PAED instrument^21^ focusing on observation of neurobehavioral symptoms. The CAPD is suited for younger children for whom assessing neurocognitive symptoms can be challenging. It includes additional cognition items and assesses all delirium subtypes across age groups. To enhance accurate screening in children under 2‐years old, developmental anchor points were provided to describe normal developmental milestones for each CAPD item.^22^ The addition of sedation assessment prior to conducting the delirium screen was added later.^19^

The SOS‐PD instrument was specifically designed to capture symptoms of paediatric delirium (PD) and iatrogenic withdrawal syndrome measured with the Sophia Observation withdrawal Symptoms scale (SOS), aiming to differentiate between the two conditions that share similar symptoms.^18^ Consisting of 22 behavioural items, 17 items represent symptoms of PD, 10 of which are also included in the SOS component. Notably, the SOS‐PD is the only instrument that incorporates parental opinion in its assessment process.

#### 5.1.3 Outcomes

Table 2 presents each instrument's original reported predictive power (sensitivity, specificity and AUC); ability to cover all delirium subtypes; age range suitability; and implementation feasibility (number of items and time to complete). The predictive power of the DSR‐88 was first undertaken in paediatric ICU in 2011; therefore, these results are included.^28^ The DRS‐88 reported the highest sensitivity and specificity levels (91.7 and 100%, respectively) while levels were acceptable and similar across other instruments. Three instruments reported the AUC^21^, ^23^, ^28^ and was highest for the CAPD (0.95 normal, 0.86 delayed development).^23^ While all instruments were designed to capture a variety of age ranges; the CAPD was the only instrument designed and tested in all age ranges.^23^ Instrument items ranged from 4 steps, or 5–22 items. The reported times to complete assessment were up to 2 min (3 pCAM‐ICU and the CAPD instruments)^20^, ^23^, ^24^, ^25^ or were not reported (DRS‐88, PAED, SOS‐PD).^18^, ^21^, ^28^

**TABLE 2:** Implementation interventions mapped against the ERIC themes.

ERIC theme	PDGUKI intervention
Engaging consumers	Parents'/patients' views were elicited in an earlier PICU research prioritization exercise (Tume), and we recruited patient support in educating healthcare professionals on the personal impact of delirium (patient video interview, PDGUKI website) PICU healthcare professionals' engagement was achieved through monthly meetings with the PDGUKI unit representatives and feedback to and from the PICUs
Using evaluative and iterative strategies	Delirium daily recording sheets, quality monitoring tools (adherence audit), delirium knowledge tests and booster training assistance was provided for unit representatives to support units to evaluate their training and progress
Changing infrastructure	Integrating the capture of daily delirium events within the PICANet database
Adapting and tailoring the context	Units adapted and tailored the mechanism of capturing daily delirium screening into existing documentation system, for example, adding screening capture to computer health records, and incorporating prompts into ward round checklists
Developing stakeholder interrelationships	Monthly PDGUKI unit representative meetings; sharing examples of unit implementation practices; encouraging delirium champions
Supporting clinicians	Prompts and reminders, for example, distribution of banner pens to bedside nurses outlining delirium screening and management, sharing resources on website
Providing interactive assistance	Local assistance and supervision
Training and educating stakeholders	Onsite training to screen and interpret the result is conducted on each PICU by trained representatives and supported by training videos on the website

Supplement, Table S1 reports the average ranks for the seven instruments based on the independent assessment of the extracted data by each reviewer. Ranks ranged from 1.25 (highest) to 6.5 (lowest). The CAPD (1.25) and SOS‐PD (1.5) ranked first and second respectively.

Currently, seven screening instruments were available to predict delirium in PICU. Two were designed for all ages in general PICUs (CAPD and SOS‐PD), three for specific age groups (pCAM‐ICU, psCAM‐ICU and sspCAM‐ICU), one that had not been tested in infants (DRS‐88), and one was designed for a post‐operative emergence delirium (PAEDS). All screening instruments were validated and reported acceptable predictive values. Reviewers ranked the CAPD and SOS‐PD instruments higher than the other instruments because they were more applicable for PICUs in the United Kingdom and Ireland that manage general and/or cardiac patients from birth to 18‐years. CAPD ranked higher than SOS‐PD for two reasons. First, CAPD had fewer items, eight versus 22, and took a shorter time to complete. Second, earlier work had established that only one PICU used the SOS instrument for predicting iatrogenic withdrawal syndrome (IWS), whereas 44% (10/23 PICUs) used an alternative IWS instrument. Adopting the SOS‐PD would necessitate changes to established IWS screening practices potentially leading to challenges.

### 5.2 Consensus results

Rapid review findings were presented during a virtual meeting of the PDUKI on 23 November 2020. PDGUKI members arranged meetings within their units to discuss their preferred instrument choice. Due to a second COVID‐19 wave in the United Kingdom and Ireland, the second PDGUKI meeting was delayed until 21 June 2021. Voting on a common screening instrument took place via Zoom (14 responses) and email for those unable to attend in person (nine responses): five PICUs did not respond. Fifteen PICUs voted for the CAPD instrument and eight for SOS‐PD. Each PICU proposed a representative to lead communication, training and implementation of delirium screening in their unit.

### 5.3 Results of training, support and dissemination

Various interventions were designed that included educational and training videos, bedside paediatric delirium screening records, posters, pre‐ and post‐training knowledge assessments with certificates, a screening audit instrument, and an online platform to monitor and provide feedback on screening adherence progress within the units. Video training materials for delirium screening were designed for the CAPD instrument, but not for the SOS‐PD instrument as its research team were developing their own training materials. All other interventions were applicable to both instruments. Monthly meetings were organized with unit training representatives to disseminate information, gather feedback and exchange best implementation practices. An openly accessible PDGUKI website was developed housing all relevant resources (https://www.qub.ac.uk/sites/uk-paediatric-delirium-group/). The training package and website was launched during a PCCS webinar open to all its UK members (8th November 2021).

### 5.4 Integrating delirium monitoring within PICANet

Discussions have been ongoing between the PDGUKI and PICANet from June 2021. A delirium descriptor was developed and added to the PICANet Web Admission Database Definitions Manual,^17^ and the database was adapted by PICANet to include delirium monitoring. The PICANet senior project manager communicated all changes with the PICANet leads at each PICU. Delirium recording was opened on the database in January 2023 for pilot testing with PICUs. In January 2024, PICANet launched collection of daily delirium events as a core activity item in the database, marking a significant step towards determining sustainability and national prevalence rates. Evaluation of successful implementation will be an ongoing process and some units have already conducted their own quality improvement evaluations. Plans to evaluate national success include monitoring the completion of delirium reporting activity by PICANet and PICU participation in the 2026 World Delirium Awareness Day (WDAD) point prevalence study.

## 6 EVALUATION OF PROGRESS

In considering successful delivery, eight of the nine themes identified in the ERIC study^12^ proved valuable as many interventions aligned with them (Table 2). The theme of ‘utilizing financial resources’ was not addressed because this initiative was undertaken without funding.

By March 2023, 6months after the launch of training, significant progress was made in implementing the plan. Eighteen of 28 PICUs (64%) successfully conducted training sessions for their staff, empowering bedside nurses to commence patient screening for paediatric delirium. On 15 March 2023, 15 PICUs (54%) chose to actively participate in the WDAD point prevalence study demonstrating a strong commitment to this initiative.^39^ The study showed that 10 of the 15 PICUs had adopted the CAPD screening instrument,^23^ while two opted for either the SOS‐PD^18^ and three for the pCAM‐ICU.^24^ This diverse selection highlights the flexibility of the strategy in accommodating the unique needs and preferences of PICUs.

## 7 DISCUSSION

A comprehensive national plan for screening paediatric delirium was implemented. The plan was methodically designed drawing upon a rapid review of current paediatric delirium screening instruments to determine a common instrument applicable for use in UK paediatric ICUs, an evidence‐based implementation strategy utilizing a multifaceted strategy and invaluable input in the decision‐making from multiprofessional staff across 28 PICUs in the United Kingdom and Ireland. The literature often lacks detailed reports on implementation planning, leaving a scarcity of examples to guide the development of effective implementation strategies for delirium screening instruments in PICUs. However, of those available, this national plan holds many similarities.

There are few existing publications of implementing paediatric delirium screening, they include: one Master's presentation, study duration 6‐months,^40^ one Doctorate of Nursing Practice study, study duration 10‐weeks^41^ and two studies, one with a 12‐week duration,^42^ and one with a 3‐year duration.^43^ Methods varied, although all studies included a passive approach of delivery using nurse education sessions. Active approaches included interrater reliability assessments nurses conducting two screening assessments,^41^ rounding and case studies,^42^ reminder text messages to bedside nurses' phones^43^ and support from champions.^40^

Their measures of success included compliance with screening using audit (all studies), pre‐ and post‐tests of knowledge on delirium and screening,^40^, ^42^, ^43^ and family satisfaction, ICU and hospital length of stay, ventilator days and cost.^40^ The studies were small and limited to single PICUs with 8 to 19 beds in the United States. All studies reported improvements at their study respective time points. Due to the varied implementation methods, resources and outcomes, findings cannot be generalized or synthesized, but offer valuable insights for those considering similar local projects.

In contrast, this paper offers a transparent and detailed account of developing and implementing a large‐scale national plan. While it lacks the detailed evaluation possible in single‐unit studies, it serves as a practical example for similar large‐scale efforts in intensive care settings. The insights into challenges and solutions encountered during nationwide implementation will be valuable for successful adoption of similar initiatives.

## 8 LIMITATIONS

A key strength of this initiative was its strong foundation in existing literature. A thorough review of relevant studies provided valuable insights into effective implementation strategies. Additionally, the impetus for change came from PICU staff rather than being imposed as a top‐down directive. This grassroots support fostered a sense of ownership and commitment to the implementation process.

There were limitations and challenges faced during the implementation of this initiative. The timing of the initiative coincided with external factors that posed significant barriers. The unprecedented COVID‐19 pandemic and the UK National Health Service (NHS) workers' strike action,^44^ created a highly strained NHS context, making the introduction of this initiative challenging. These external factors likely impacted the implementation process and its outcomes.

A further constraint was lack of funding for the initiative. During the implementation period, most of the UK NHS funding was directed towards COVID‐19 research. Consequently, we were unable to secure research funding for data collection in individual PICUs, such as pre and post knowledge scores, training targets and screening adherence audits. These data would have allowed for a more thorough evaluation of the implementation plan.

Despite its limitations, the implementation plan aimed to address the challenges and work within available means for collecting data, such as participating in the WDAD study and integrating delirium data collection into the PICANet national registry. Looking ahead, individual units should consider financial resources to sustain staff training and support in the future.

## 9 IMPLICATIONS FOR PRACTICE

Implementing delirium screening presents numerous opportunities that can significantly enhance the quality of care in PICUs. By incorporating daily screening into the routine practices of all PICUs, trends in delirium incidence can be effectively monitored over time. This approach will be particularly valuable when assessing the efficacy of future interventions designed to mitigate delirium. In the context of UK and Ireland PICUs, family‐centred care with open visiting policies promotes an active and influential parental role on PICU. This increased involvement equips parents with a unique opportunity to detect early indicators of abnormal reactions which may signal the onset of delirium. It is essential that nurses use parents' experience of their child in assessing for delirium.

Nonetheless, it is important to recognize and address challenges that clinical staff may encounter when initiating and sustaining delirium screening over time. Challenges include behaviour maintenance strategies outlined by Kwasnicka and colleagues.^45^ Educating staff on the positive outcomes of delirium screening will boost motivation. Equipping staff with skills and resilience will foster control and enhance sustainability of delirium screening. Comprehensive, ongoing multidisciplinary training and support for bedside nurses are essential for success. Supportive influences from the intensive care community, including parents, are crucial for maintaining screening practice. Trust and respect are key for adherence; hence, engaging influential figures and parents is important. Moreover, nurturing collegiality among PICU staff and working towards a common purpose, can motivate adherence. Addressing these challenges proactively can successfully integrate delirium screening in PICUs. This strategic approach can enhance patient outcomes and quality of care provided in the PICU.

## 10 CONCLUSION

This quality improvement initiative addressed the recommendations from international clinical guidelines for daily screening of delirium in critically ill children in intensive care. We provided clinicians with validated screening tools for detecting paediatric delirium and the necessary support and infrastructure to maintain screening. Additionally, the initiative included critical steps for maintaining practice of delirium screening in PICUs by embedding screening in the national PICANet database. Notwithstanding, embedding and sustaining screening is an ongoing challenge.

## AUTHOR CONTRIBUTIONS

All authors made equal and substantial contributions to the following: (1) the conception and design of the initiative, (2) drafting the article or revising it critically for important intellectual content and (3) final approval of the version to be submitted. BB, LA, AL and LT were responsible for the rapid review. BB, JC, MM, LMcI, SG‐P and LT were responsible for the training resources. LMcI developed the website.

## CONFLICT OF INTEREST STATEMENT

The authors have no conflicts of interest.

## ETHICS STATEMENT

Ethical approval was not required for this initiative. Units that participated in the World Delirium Day Awareness point prevalence study obtained their institutional clinical audit office approvals.

## Supporting information


**Data S1.** Supporting information.

## Data Availability

The data that support the findings of this study are available from the corresponding author upon reasonable request.
