# Supplementary material for: National screening for delirium in paediatric intensive care units: A quality improvement initiative
Source: Nurs Crit Care. 2025 Feb 24;30(2):e13303. doi: 10.1111/nicc.13303 (PMC11848506; doi:10.1111/nicc.13303)
Supplement: Supplementary file 1 — Data S1. Supporting information. [file NICC-30-0-s001.docx]

# Supplementary Material for ‘National screening for delirium in Paediatric Intensive Care Units: a quality improvement initiative’.

## Contents

Pages 2-3 Rapid Review Search Strategy

Page 3 Table 1 Rapid Review results in rank order

## Rapid Review Search Strategy

Database(s): Ovid MEDLINE(R) ALL 1946 to August 2020

1 Critical Care/

2 intensive care.mp.

3 Infant/ or Child/ or paediatric.mp.

4 delirium.mp. or Delirium/

5 screening tools.mp.

6 screening instruments.mp.

7 delirium detection.mp.

8 Intensive Care Units, Pediatric/ or Critical Illness/

9 assessment tool.mp.

10 Psychometrics/ or scale development.mp.

11 psychometric evaluation.mp.

12 reliability.mp.

13 validity.mp.

14 rating scale.mp.

15 item reduction.mp.

16 "Systematic Review"/

17 1 or 2 or 8

18 5 or 6 or 9 or 10 or 11 or 12 or 13 or 14 or 15

19 3 and 4 and 17 and 18

**CINAHL**

S10 AB ( pediatric delirium or paediatric delirium ) AND ( screening or assessment or test or diagnosis )

S9 AB ( pediatric delirium or paediatric delirium ) AND ( screening or assessment or test or diagnosis )

S8 S1 AND S4 AND S6 AND S7

S7 S2 OR S3

S6 S2 OR S5

S5 "delirium in the intensive care unit"

S4 "paediatric or pediatric or children or child or infant or young person"

S3 (MH "Psychometrics") OR (MH "Reliability and Validity") OR (MH "Predictive Validity") OR (MH "Internal Validity") OR (MH "Instrument Validation")

S2 "delirium screening"

S1 (MH "Intensive Care Units, Pediatric")

**PubMed** searches included key words taken from the above databases.

Table 1. Rapid review results in rank order

| **Paediatric Delirium Screening Tools** | **Reviewer 1** | **Reviewer 2** | **Reviewer 3** | **Reviewer 4** | **Average rank** |
| --- | --- | --- | --- | --- | --- |
| Cornell Assessment of Pediatric Delirium (CAPD) | 1 | 1 | 1 | 2 | 1.25 |
| Sophia Observation withdrawal Symptoms-Paediatric Delirium scale (SOS-PD) | 2 | 1 | 2 | 1 | 1.5 |
| Preschool CAM-ICU (psCAM-ICU) | 3 | 3 | 3 | 3 | 3 |
| Pediatric Confusion Assessment Method (pCAM-ICU) | 3 | 3 | 3 | 4 | 3.25 |
| Severity scale for the pCAM-ICU (sspCAM-ICU) | 6 | 5 | 5 | 5 | 5.25 |
| Pediatric Anesthesia Emergence Delirium scale (PAED) | 5 | 6 | 6 | 6 | 5.75 |
| Delirium Rating Scale (DRS) | 6 | 7 | 7 | 6 | 6.5 |
